# Costs of care at the end of life among elderly patients with chronic kidney disease: patterns and predictors in a nationwide cohort study

**DOI:** 10.1186/s12882-017-0456-2

**Published:** 2017-01-26

**Authors:** Bradley Chen, Victoria Y. Fan, Yiing-Jenq Chou, Chin-Chi Kuo

**Affiliations:** 1 Institute of Public Health, National Yang-Ming University, Taipei, Taiwan; 2 Department of Public Health Sciences & Epidemiology, University of Hawaii at Manoa, 1960 East-West Road, Biomed D204 Honolulu, HI USA; 3 François-Xavier Bagnoud Center for Health and Human Rights, Harvard T.H. Chan School of Public Health, 651 Huntington Ave, Boston, MA USA; 4 Center for Global Development, Washington, D.C., USA; 5 Institute of Hospital and Health Care Administration, National Yang-Ming University, Taipei, Taiwan; 6 Big Data Center, China Medical University Hospital, Taichung, Taiwan; 7 Kidney Institute and Division of Nephrology, Department of Internal Medicine, China Medical University Hospital and College of Medicine, China Medical University, 13F.-2, No.101, Kaixuan Rd., East Dist, Tainan City, Taiwan

**Keywords:** End-of-life care, Chronic kidney disease, Health care costs, Intensive procedures, Elderly

## Abstract

**Background:**

Despite the urgent need for evidence to guide the end-of-life (EOL) care for patients with chronic kidney disease (CKD), we have limited knowledge of the costs and intensity of EOL care in this population. The present study examined patterns and predictors for EOL care intensity among elderly patients with CKD.

**Methods:**

We conducted a retrospective nationwide cohort study utilizing the Taiwan National Health Insurance (NHI) Research Database. A total of 65,124 CKD patients aged ≥ 60 years, who died in hospitals or shortly after discharge between 2002 and 2012 were analyzed. The primary outcomes were inpatient expenses and use of surgical interventions in the last 30 days of life. Utilization of intensive care unit (ICU), mechanical ventilation, resuscitation, and dialysis was also examined in a sub-sample of 2072 patients with detailed prescription data. Multivariate log-linear and logistic regression analyses were performed to assess patient-, physician-, and facility-specific predictors and the potential impact of a 2009 payment policy to reimburse hospice care for non-cancer patients.

**Results:**

During the last 30 days of life, average inpatients costs for elderly CKD patients were approximately US$10,260, with 40.9% receiving surgical interventions, 40.2% experiencing ICU admission, 45.3% undergoing mechanical ventilation, 14.7% receiving resuscitation and 42.0% receiving dialysis. Significant variability was observed in the inpatient costs and use of intensive services. Costs were lower among individuals with the following characteristics: advanced age; high income; high Charlson Comorbidity Index scores; treatment by older physicians, nephrologists, and family medicine physicians; and treatment at local hospitals. Similar findings were obtained for the use of surgical interventions and other intensive services. A declining trend was detected in the costs of EOL care, use of surgical interventions and resuscitation between 2009 and 2012, which is consistent with the impact of a 2009 NHI payment policy to reimburse non-cancer hospice care.

**Conclusions:**

Overall EOL costs and rates of intensive service use among older patients with CKD were high, with significant variability across various patient and provider characteristics. Several opportunities exist for providers and policy makers to reduce costs and enhance the value of EOL care for this population.

## Background

Support for reforming the delivery of end-of-life (EOL) care has been observed in recent years, exemplified by the 2014 Institute of Medicine Report “Dying in America” [1]. Several policy interventions have also been suggested, including the recently proposed regulations by the Centers for Medicare and Medicaid Services to reimburse physicians for holding advance care planning discussions with patients [2]. In Taiwan, the government launched a pilot program in 2011 and amended its Statute of Palliative Care in 2014 to promote hospice and palliative care [3]. However, the current practice of EOL care for these patients, including most of the well-intentioned programs and initiatives to improve quality or reduce costs, is not evidence-based [4].

In the past decade, several studies have examined the intensity of EOL care, revealing significant variation in treatment practices [5–12]. To date, most of our understanding of the determinants of EOL care intensity has largely ensued from cancer care. Relevant investigations specific to other diseases, including chronic kidney disease (CKD), are relatively much fewer [13–15]. Meanwhile, an improvement in EOL care for patients with CKD is particularly needed [16, 17] because of not only the surging prevalence and incidence of CKD and end-stage renal disease (ESRD) worldwide [18, 19], but also the remarkable rise in inpatient care for patients with ESRD during their final months [20]. Several studies have reported that patients with CKD experience substantial disease burdens at the end of life [21, 22], and that their needs and preferences are not integrated with their care [23]. The intensity of EOL care for patients with ESRD could be higher than that observed for patients with cancer, heart failure, or dementia [24, 25].

However, with the ongoing efforts to optimize EOL care for patients with CKD and ESRD [16, 23, 26–28], emerging literature on the determinants of care for this population has concentrated on patient characteristics, particularly their demographics and regional and temporal variation [15, 24, 29–31]. Information on the patterns of EOL care intensity across physician and hospital organizational determinants is scarce. Moreover, limited evidence exists regarding the impact of payment policies targeting EOL care. Understanding these nonclinical factors can enable providers and policy makers to improve the quality of care among this growing and vulnerable population.

To fill the knowledge gaps, we used the inpatient claims database of the National Health Insurance (NHI) program in Taiwan to conduct a systematic examination of the costs of EOL care among patients with CKD from 2002 to 2012. In addition, we investigated a number of commonly reported measures of intensive services. EOL spending has been shown to reflect the differences in the intensity of care across health systems [32, 33] and is of great policy interest. Medical spending also has grave implications financially and for the well-being of individuals and households [34]. Our study had the advantages of universal population coverage and the generous and unified NHI benefit package; these factors avoided the bias driven by disparities in access and patient incentives, which are commonly encountered limitations of studies involving a multiple-choice plan or multi-payer system.

## Methods

### Study population

To assess the costs of EOL care among patients with CKD, we employed a retrospective cohort design and identified a cohort of CKD decedents whose enrollment in the NHI program was terminated because of death and who received a primary or secondary diagnosis of CKD (International Classification of Disease, Version 9, Clinical Modification codes:585.1–585.9) during inpatient visits during the last year of their life. The identified decedents represented CKD patients who passed away in hospitals or shortly after discharge. The latter is because of the cultural image of “dying at home” in Taiwan, which results in a common practice to discharge dying patients so they can return home to die [35]. Because of the rapid increase in the CKD disease burden among the elderly population, only decedents whose age at death was ≥ 60 years were included in the analyses.

### Data sources

Data were obtained from the Taiwan National Health Insurance Research Database (NHIRD), which contains the complete claim records for the entire insured population [36]. Taiwan’s NHI program covers more than 99% of the population, and under the generous benefit package, patients can readily access any provider of choice with limited copayment and co-insurance [37]. We constructed our longitudinal data from two specific inpatient databases. The primary cohort was the inpatient expenditure summary by admission, for the entire population from 2002 to 2012. The inpatient expenditure summary files provided data for each admission, including the principal and secondary diagnoses, length of stay, total charge, and the expenditure breakdown according to categories, such as surgical, examination, drug expenses, and etc. To explore the detailed inpatient services provided, we also accessed a second data source, containing the complete inpatient prescriptions and orders of 1,000,000 randomly sampled individuals (out of the approximately 25,700,000 beneficiaries in the 2005 registry) [38]. This database provides detailed accounts of health service utilization for sampled decedents (secondary cohort); however, because the sampling was based on beneficiaries in 2005, only information of decedents between 2005 and 2012 is available.

### Primary and secondary outcomes

Because EOL care, including hospice care, in Taiwan often occurs in a hospital rather than a home or nursing home [39], the main outcome in our study was inpatient expenditures during the last 30 days of life, which summarizes the intensity and utilization of health resources during the last stage of life. We used nominal expenditures in New Taiwan Dollars ($NT) because the reimbursement of services in Taiwan is based on a fee schedule that is not updated for inflation. Measures of specific health services in the literature of EOL care intensity typically fall into one of the following three categories: hospitalization [e.g., acute care and intensive care unit (ICU) use], life-sustaining interventions (e.g., resuscitation and dialysis), and potentially life-prolonging treatments (e.g., surgery and chemotherapy) [13]. Therefore, we assessed the aggressiveness of EOL care through binary variables, which indicated whether in the last month of life, patients were admitted to the ICU; received mechanical ventilation, resuscitation, or dialysis treatments (for decedents between 2005 and 2012, secondary cohort); or underwent any surgical interventions (for decedents between 2002 and 2012, primary cohort).

### Patient characteristics

Patient characteristics assessed as predictors of intensity of care were age; sex; beneficiary’s earnings, on the basis of which the payroll-based premium is calculated; and co-morbidities, as defined by the Charlson Comorbidity Index (CCI) [40]. We also considered whether the patient had ESRD requiring renal replacement therapy, because of the expansive nature of dialysis treatments. In addition, patients with ESRD in Taiwan are entitled to free medical services without any patient cost-sharing under NHI, which could affect their utilization of health services. Eligibility for such a medical fee waiver is indicated in the NHIRD.

### Provider characteristics

The primary attending physician and hospital were defined respectively as the doctor and hospital that accounted for the largest EOL expense during the final month of the patients’ lives. We identified providers with a major role in shaping the intensity of EOL care. The Taiwanese medical profession is highly specialized, and different specialties typically operate their own separate wards in the hospitals. The NHIRD includes anonymous physician characteristics including age, sex, and the specialty of the primary physician (i.e., whether they were surgeons, nephrologists, family medicine physicians, or other types of physicians). Family medicine physicians were identified because hospice and palliative care and associated fellowship training are often affiliated with family medicine in Taiwan.

We also examined the role of the facility characteristics of the treating hospitals, including ownership (public and private), accreditation (medical center, regional, or local hospital, according to facility capacity in terms of bed numbers, medical specialties, and staff densities), and teaching status.

### Regional determinants

The hospital sector in Taiwan is organized into six regions, each with its own capped budget, under which hospitals within the same region compete for reimbursement. We also introduced year-specific number of hospitals and beds in the region to explore the role of market consolidation and mergers. Finally, our model also included region dummies to control for any effects from unobservable regional-level factors.

### Policy impact

Health systems are rarely static and frequently reacting to policy changes. Specifically, in 2009, NHI　program implemented a payment policy that reimbursed providers for non-cancer EOL hospice care [3]. We explored the impact of this policy by performing a spline regression to test whether a difference existed in the time trends of costs or intensity of EOL care before and after 2009. That is, in addition to the “*year”* variable that reflects the linear time trend of cost or intensity, a separate term, “*year-2009*”, was introduced to capture the change in time trend after 2009.

### Statistical analysis

To assess the variability in EOL care, the average inpatient expenses and probability of receiving intensive care, including ICU stay, mechanical ventilation, resuscitation, dialysis and any surgical interventions, in the last 30 days of life were estimated separately for various stratified sub-groups. Bivariate associations between costs and predictors were statistically tested using *t-*tests and ANOVA. Chi-squared tests were used to compare the binary data for intensive care across sub-groups.

For the analysis of the costs of care, inpatient costs were first log-transformed to satisfy normality requirements (Appendix 1). We used multivariable log-linear models coupled with spline regression to estimate the effects of patient, physician, facility characteristics, regional factors, and the potential policy impact on the costs of care during the final month of life. For easier interpretation of the log-linear model, we calculated the exponentiated coefficients, which represented the ratios of the geometric mean of expenses relative to those of the reference group. Multivariable logistic models were used for the analysis of intensive care received at the end of life, and odds ratios (ORs) of respective predictors were estimated. A *p* value of less than .05 was considered statistically significant. All models used robust standard errors, with adjustment for patient clustering within a particular hospital. All analyses were performed using Stata, version 13.1 (StataCorp LP., College Station, Texas, USA).

## Results

From 2002 to 2012, a total of 65,124 CKD decedents aged ≥ 60 years who passed away in hospitals or shortly after discharge were identified. The mean (SD) age at death was 77.5 (8.7) years and 42.9% had a diagnosis of ESRD (Table 1). During the last 30 days of life, 23.9% of decedents received inpatient care from nephrologists, with an additional 12.9% receiving care from surgeons, 4.1% from family medicine physicians, and the remaining patients from physicians of other specialties. Regional hospitals accounted for the largest share (43.5%) of EOL inpatient care, followed by medical centers (33.6%); the majority of inpatient care took place in private hospitals (66.5%). Across regions, Taipei had the largest number of the CKD decedents (28.8%), whereas the eastern region had the fewest (3.6%). During the study period, the annual number of CKD decedents peaked at 7164 during 2003, when Taiwan experienced an outbreak of severe acute respiratory syndrome that may have caused adverse health outcomes [41]. Through the database of 1,000,000 randomly sampled individuals, we had access to the detailed prescription data of 2072 decedents between 2005 and 2012. The characteristics of this sub-sample are similar to those of the population-based sample (Table 1).

**Table 1:** Characteristics of study cohorts of chronic kidney disease decedents dying in hospitals in Taiwan, 2002–2012

Characteristics	Population-based primary cohort (2002–2012)	Randomly sampled secondary cohort (2005–2012)
	(*n* = 65,124)	(*n* = 2072)
Age at death, NO. (%)		
60–70	13,136 (20.2%)	374 (18.1%)
70–80	24,086 (37.0%)	749 (36.2%)
80+	27,902 (42.8%)	949 (45.8%)
Age at death, mean (SD)	77.5 (8.7)	78.1 (8.6)
Insurable earnings, mean (SD)	7498.8 (10,649.3)	7448.9 (10,608.1)
ESRD, NO. (%)	27,911 (42.9%)	913 (44.1%)
Charlson Comorbidity Index, mean (SD)	3.7 (1.8)	3.7 (1.8)
Specialty of Primary Physician (%)		
Surgeon	8428 (12.9%)	275 (13.3%)
Nephrologist	15,559 (23.9%)	431 (20.8%)
Family Medicine	2642 (4.1%)	80 (3.9%)
Others	38,495 (59.1%)	1286 (62.1%)
Primary Hospital- Accreditation, NO. (%)		
Medical Centers	21,856 (33.6%)	706 (34.1%)
Regional Hospitals	28,341 (43.5%)	900 (43.4%)
Local Hospitals	14,927 (22.9%)	466 (22.5%)
Primary Hospital- Ownership, NO. (%)		
Public	21,850 (33.6%)	745 (36.0%)
Private	43,274 (66.5%)	1327 (64.0%)
Region, NO. (%)		
Taipei (Capital)	18,746 (28.8%)	645 (31.1%)
Northern	9911 (15.2%)	307 (14.8%)
Central	12,668 (19.5%)	392 (18.9%)
Southern	10,401 (16.0%)	318 (15.4%)
Kao-Ping	11,058 (17.0%)	331 (16.0%)
Eastern	2340 (3.6%)	79 (3.8%)
Year of death, NO. (%)		
2002	4989 (7.7%)	-
2003	7164 (11.0%)	-
2004	6917 (10.6%)	-
2005	6236 (9.6%)	263 (12.7%)
2006	6156 (9.5%)	264 (12.7%)
2007	5934 (9.1%)	276 (13.3%)
2008	5747 (8.8%)	290 (14.0%)
2009	5295 (8.1%)	242 (11.7%)
2010	5544 (8.5%)	239 (11.5%)
2011	5289 (8.1%)	243 (11.7%)
2012	5853 (9.0%)	255 (12.3%)

### Variation of intensity in end-of-life care

Table 2 presents the distribution of our outcome measures, namely 30-day EOL inpatient expenses and the probability of receiving intensive health services such as surgical interventions, ICU admission, mechanical ventilation, resuscitation, and dialysis treatment. The average 30-day EOL inpatient expense was approximately US$10,260 (NT$332,422, at US$1 = NT$32.4) and varied from US$8000 (NT$260,000) to US$12,300 (NT$400,000). Expenses were relatively higher for patients aged 60–70 years, females with lower insurable earnings, and those with ESRD; in addition, expenses incurred at medical centers and public hospitals were also higher. Moreover, apparent temporal variations were observed. All the aforementioned differences were statistically significant (Table 2). The variation in the average probabilities of receiving surgical interventions across sub-populations was generally similar to that of the EOL inpatient expenses, except that the difference in the probabilities of surgical interventions by insurable monthly earnings group was not statistically significant.

**Table 2:** Bivariate associations between selected predictors and end-of-life care intensity measures among Taiwanese CKD decedents dying in hospitals, 2002–2012

Characteristics	Expenses (NT$^a^), Mean (SD)	p-value^b^	Surgical Interventions, %	p-value^c^	ICU Use, %	p-value^c^	Mechanical Ventilation, %	p-value^c^	Resuscitation, %	p-value^c^	Dialysis, %	p-value^c^
	(*n* = 65,124)		(*n* = 65,124)		(*n* = 2072)		(*n* = 2072)		(*n* = 2072)		(*n* = 2072)	
Overall	332,422 (413,204)	-	40.9%	-	40.2%	-	45.3%	-	14.7%	-	42.0%	-
Age at death												
60–70	346,843 (438,247)	<0.001	45.9%	<0.001	40.4%	0.16	46.5%	0.80	20.3%	<0.001	48.4%	<0.001
70–80	340,080 (412,106)		43.5%		42.7%		45.5%		15.6%		47.5%	
80+	319,024 (401,455)		36.3%		38.1%		44.6%		11.7%		35.2%	
Sex												
Male	328,808 (411,335)	0.01	39.6%	<0.001	39.3%	0.34	44.5%	0.43	14.8%	0.82	39.1%	0.003
Female	336,754 (415,398)		42.5%		41.3%		46.2%		14.5%		45.7%	
Insurable monthly earnings												
Below average ($NT7,499)	348,894 (426,698)	<0.001	41.2%	0.08	39.7%	0.48	47.1%	0.02	15.4%	0.18	42.9%	0.25
Average and above	300,096 (383,307)		40.5%		41.3%		41.6%		13.2%		40.3%	
ESRD												
Yes	369,902 (441,971)	<0.001	47.7%	<0.001	47.8%	<0.001	51.2%	<0.001	19.8%	<0.001	67.5%	<0.001
No	304,312 (387,875)		35.9%		36.3%		40.6%		10.6%		22.0%	
Primary Hospital- Accreditation												
Medical Centers	418,779 (492,098)	<0.001	49.9%	<0.001	38.5%	0.13	43.6%	0.10	12.3%	0.01	45.0%	0.14
Regional Hospitals	294,439 (361,834)		41.5%		42.7%		44.3%		14.4%		40.7%	
Local Hospitals	278,099 (353,787)		26.7%		38.0%		49.6%		18.7%		40.1%	
Primary Hospital												
Public	388,234 (434,996)	0.01	41.6%	0.01	38.7%	0.28	42.8%	0.09	15.3%	0.54	40.0%	0.16
Private	329,489 (401,725)		40.6%		41.1%		46.6%		14.3%		43.2%	
Year of death												
2002	262,495 (329,490)	<0.001	40.7%	<0.001	-		-		-		-	
2003	300,027 (377,547)		41.1%		-		-		-		-	
2004	348,311 (437,586)		42.8%		-		-		-		-	
2005	344,680 (422,834)		40.4%		44.9%	0.04	51.3%	0.01	16.3%	0.02	45.2%	0.22
2006	333,470 (413,808)		40.8%		40.9%		45.5%		18.2%		35.6%	
2007	352,703 (423,098)		42.3%		43.1%		50.7%		17.0%		44.6%	
2008	354,350 (439,043)		42.2%		45.5%		49.3%		16.6%		44.5%	
2009	353,732 (414,014)		43.2%		35.5%		42.1%		15.7%		42.6%	
2010	341,921 (423,078)		40.0%		38.9%		40.2%		13.8%		43.9%	
2011	334,486 (405,408)		39.1%		33.3%		42.8%		10.3%		37.4%	
2012	326,514 (430,010)		37.2%		37.6%		38.4%		8.6%		42.0%	

a. NT$ = New Taiwan Dollars; US$1 = NT$32.4

b. p-value of *t*-test for binary variables and ANOVA for variables with multiple categories

c. p-value of chi-squared test

In the secondary cohort of 2072 CKD decedents, 40.2% were admitted to the ICU, 45.3% received mechanical ventilation, 14.7% underwent resuscitation, and 42.0% received dialysis treatment during the final month of life. These intensive services were relatively more common among those with ESRD. Resuscitation and dialysis treatments were more common among patients aged 60–70 years than those aged >70 years (Table 2).

### Predictors of end-of-life medical expenses

The results of the multivariate log-linear regression models are shown in Table 3. The exponentiated coefficients provide easier interpretation of the variation in the costs of care. For instance, the ratio of the geometric mean inpatient expenses among ESRD decedents to that of the non-ESRD group was 1.29 [95% confidence interval (CI), 1.24–1.33], implying that the costs were 29% (95% CI, 24–33%) higher among ESRD decedents. The fully adjusted estimates indicated that the costs of care were higher among females (3%; 95% CI, 0–5%) and lower among patients with advanced ages: every 10-year increase in age was associated with a 3% (95% CI, 2–5%) reduction in 30-day EOL inpatient expenses. In addition, increased co-morbidities were associated with lower EOL care costs, with each unit increase in CCI score being associated with a 3% (95% CI, 2–4%) drop. EOL expenses were also 13% (95% CI, 10–16%) lower among individuals with above average monthly earnings.

**Table 3:** Determinants of inpatient costs and use of surgical interventions among Taiwanese CKD decedents dying in hospitals, 2002–2012 (*N* = 65,124)^a^

Characteristics		End-of-life Inpatient Costs		Surgical Interventions	
		Exponentiated Coefficients	95% Confidence Interval	Odds Ratios	95% Confidence Interval
Patient	Age at death (in tens, '0)	0.97***	(0.95–0.98)	0.88***	(0.86–0.90)
	Sex (=female)	1.03*	(1.00–1.05)	1.10***	(1.07–1.14)
	ESRD	1.29***	(1.24–1.33)	1.33***	(1.25–1.42)
	Insurable monthly earnings (=average and above)	0.87***	(0.84–0.90)	0.94**	(0.90–0.99)
	Charlson Comorbidity Index (CCI)	0.97***	(0.96–0.98)	0.93***	(0.91–0.94)
Physician	Physician age (in tens, '0)	0.94***	(0.91–0.97)	0.86***	(0.81–0.91)
	Physician sex (=female)	0.94	(0.87–1.02)	0.91	(0.82–1.01)
	Specialty of primary attending physician				
	Nephrologist	0.74***	(0.70–0.78)	1.37***	(1.26–1.49)
	Family medicine	0.62***	(0.55–0.71)	0.60***	(0.48–0.75)
	Surgeon	1.11*	(1.02–1.21)	3.56***	(3.20–3.97)
Facility	Primary hospital- accreditation level				
	Medical Centers	1.59***	(1.41–1.80)	1.88***	(1.65–2.15)
	Regional Hospitals	1.17**	(1.06–1.28)	1.40***	(1.24–1.59)
	Local Hospitals (Ref.)	-	-	-	-
	Primary hospital- ownership (=private)	1.06	(0.98–1.14)	1.06	(0.96–1.16)
	Teaching hospital	0.96	(0.85–1.08)	1.43***	(1.22–1.69)
Region	Number of hospital in the region (in tens, '0)	1.02	(0.98–1.06)	1.02	(0.98–1.07)
	Number of beds in the region (in hundreds, '00)	1.00	(1.00–1.00)	1.00	(1.00–1.00)
Time Trend	Overall time trend (Year)	1.04***	(1.02–1.05)	1.02	(1.00–1.05)
	Difference in trend post 2009 (=Year-2009, for year ≥ 2009)	0.91***	(0.89–0.93)	0.91***	(0.87–0.96)

Standard errors are clustered at the hospital level

*** *p* < 0.001, ** *p* < 0.01, * *p* < 0.05

a. Estimates are adjusted for regional fixed effects by including regional dummies

Provider characteristics, both at the physician and hospital levels, had significant and substantial impacts on the EOL expenses incurred. Older physicians were associated with lower costs for EOL care: every 10-year increase in physician age was associated with a reduction in the 30-day EOL inpatient expenses by 6% (95% CI, 3–9%). Those who received care from surgeons had 11% (95% CI, 2–21%) higher costs. By contrast, decedents treated by nephrologists incurred 26% (95% CI, 22–30%) lower costs during EOL care. With a 38% (95% CI, 29–45%) reduction, the costs were even lower for treatment by family medicine physicians, compared with the costs for those treated by clinicians of other non-surgical specialties. At the hospital level, assuming that all other variables were constant, the EOL care costs for patients with CKD were much higher at medical centers and regional hospitals, with 59% (95% CI, 41–80%) and 17% increases (95% CI, 6–28%), respectively, compared with the costs incurred at local hospitals. Hospital ownership and teaching status, as well as the regional supply in the number of hospitals or beds, did not have a statistically significant effect on the EOL care costs.

During the study period, a trend of increasing EOL expenses, with an annual increase of 4% (95% CI, 2–5%), was also observed. However, after 2009, a divergence in the trend was noted, with an annual reduction of 9% (95% CI, 7–11%) in EOL inpatient costs.

### Odds of receiving intensive services

Table 3 also presents the OR estimates of receiving any surgical intervention during the last 30 days of life, and the results were consistent with the findings on inpatient expenses. Older CKD decedents were less likely to have received surgical interventions if they were male, had a more advanced age, did not have ESRD, had more co-morbidities, had a higher income, were attended to by older physicians, or were treated at local hospitals. However several notable differences were observed. First, the odds of receiving surgical interventions increased for decedents treated by nephrologists (OR = 1.37; 95% CI, 1.26–1.49). By contrast, patients with CKD seen by family medicine physicians had much lower odds (OR = 0.60; 95% CI, 0.48–0.75) of undergoing surgical interventions during EOL care than did those seen by other physicians with non-surgical clinical specialties. Second, CKD decedents in teaching hospitals were more likely to have received surgical interventions (OR = 1.43; 95% CI, 1.22–1.69). Third, no overall statistically significant trend of increasing odds of receiving surgical interventions at the end of life was observed between 2002 and 2009; nevertheless, a declining trend in the odds of receiving any surgical interventions after 2009 (OR = 0.91, 95% CI, 0.87–0.96) was noted.

Analyses of the sub-sample with detailed prescription data are summarized in Table 4. To confirm the robustness, identical analyses of the costs and surgical interventions in the population-based data were performed. The results were consistent, with predictors for the costs of care including age at death, earnings, and CCI scores, of which the coefficients were identical, but the statistical significance was lost because of the small sample size.

**Table 4:** Determinants of costs and intensive service use among randomly sampled Taiwanese CKD Decedents dying in hospitals, 2005–2012 (*N* = 2072)^a^

Characteristics		End-of-life Inpatient Costs		Surgical Interventions		ICU		Mechanical Ventilation		Resuscitation		Dialysis	
		Exponentiated Coefficients	95% Confidence Interval	Odds Ratios	95% Confidence Interval	Odds Ratios	95% Confidence Interval	Odds Ratios	95% Confidence Interval	Odds Ratios	95% Confidence Interval	Odds Ratios	95% Confidence Interval
Patient	Age at death (in tens, '0)	0.97	(0.91–1.03)	0.91	(0.81–1.02)	0.98	(0.88–1.09)	0.97	(0.87–1.09)	0.78**	(0.66–0.91)	0.97	(0.85–1.10)
	Sex (=female)	1.00	(0.91–1.11)	1.05	(0.87–1.28)	1.02	(0.83–1.24)	1.03	(0.85–1.25)	0.89	(0.70–1.13)	1.07	(0.86–1.32)
	ESRD	1.25***	(1.12–1.40)	1.23	(1.00–1.53)	1.74***	(1.38–2.21)	1.54***	(1.24–1.90)	1.97***	(1.52–2.55)	6.52***	(5.22–8.15)
	Insurable monthly earnings (=average and above)	0.88	(0.78–1.00)	0.82	(0.67–1.00)	1.06	(0.86–1.31)	0.80*	(0.66–0.96)	0.84	(0.64–1.10)	0.88	(0.69–1.11)
	Charlson Comorbidity Index (CCI)	0.97	(0.95–1.00)	0.92***	(0.87–0.96)	0.84***	(0.79–0.90)	0.86***	(0.81–0.91)	0.86***	(0.79–0.93)	0.99	(0.94–1.05)
Physician	Physician age (in tens, '0)	0.90**	(0.83–0.97)	0.81**	(0.71–0.92)	0.85*	(0.73–0.99)	0.96	(0.82–1.11)	0.98	(0.83–1.16)	0.89	(0.77–1.02)
	Physician sex (=female)	1.01	(0.85–1.20)	0.85	(0.60–1.20)	0.90	(0.59–1.37)	0.89	(0.60–1.32)	0.73	(0.42–1.26)	1.48	(0.98–2.25)
	Specialty of primary attending physician												
	Nephrologist	0.82**	(0.71–0.95)	1.54***	(1.23–1.93)	0.87	(0.66–1.14)	0.84	(0.65–1.09)	1.03	(0.75–1.43)	1.73***	(1.30–2.30)
	Family medicine	0.39***	(0.27–0.57)	0.74	(0.38–1.44)	0.58	(0.32–1.04)	0.35***	(0.19–0.62)	0.77	(0.37–1.59)	0.56	(0.31–1.02)
	Surgeon	1.22*	(1.02–1.46)	4.31***	(3.20–5.83)	1.30	(0.94–1.78)	1.01	(0.77–1.31)	1.20	(0.76–1.90)	1.19	(0.89–1.58)
Facility	Primary hospital- accreditation level												
	Medical Centers	1.68***	(1.30–2.15)	2.01**	(1.27–3.19)	0.93	(0.65–1.33)	0.93	(0.63–1.36)	0.65	(0.40–1.08)	1.51	(0.95–2.40)
	Regional Hospitals	1.23	(0.96–1.56)	1.83**	(1.17–2.87)	1.18	(0.85–1.62)	0.99	(0.71–1.38)	0.88	(0.54–1.41)	1.18	(0.76–1.84)
	Local Hospitals (Ref.)	-	-	-	-	-	-	-	-	-	-	-	-
	Primary hospital- ownership (=private)	1.11	(0.99–1.26)	1.03	(0.83–1.28)	1.10	(0.89–1.35)	1.12	(0.90–1.40)	0.79	(0.61–1.04)	1.05	(0.83–1.32)
	Teaching hospital	0.80	(0.61–1.06)	1.24	(0.74–2.08)	0.93	(0.63–1.38)	0.71	(0.48–1.05)	0.74	(0.43–1.26)	0.62	(0.38–1.03)
Region	Number of hospital in the region (in tens, '0)	1.11	(0.93–1.33)	0.98	(0.74–1.29)	1.03	(0.74–1.43)	1.10	(0.84–1.44)	1.16	(0.77–1.75)	1.05	(0.75–1.45)
	Number of beds in the region (in hundreds, '00)	1.00	(0.99–1.01)	1.00	(0.99–1.01)	1.00	(0.99–1.01)	0.99	(0.99–1.00)	0.99	(0.98–1.01)	1.00	(0.99–1.01)
Time Trend	Overall time trend (Year)	1.07	(0.98–1.16)	1.12	(0.98–1.28)	0.92	(0.80–1.06)	0.97	(0.86–1.10)	1.06	(0.88–1.26)	1.00	(0.85–1.16)
	Difference in trend post 2009 (=Year-2009, for year ≥ 2009)	0.87*	(0.78–0.97)	0.75**	(0.61–0.91)	1.05	(0.87–1.27)	0.97	(0.83–1.15)	0.76*	(0.59–0.99)	0.97	(0.78–1.21)

Standard errors are clustered at the hospital level

*** *p* < 0.001, ** *p* < 0.01, * *p* < 0.05

a. Estimates are adjusted for regional fixed effects by including regional dummies

Our multivariable logistic models showed that the predictors for ICU admission, mechanical ventilation and resuscitation were similar: the odds among patients with ESRD were high, and among those with more co-morbidities and those seen by family medicine clinicians the odds were lower. In addition, older physicians were associated with lower odds of ICU stay (OR = 0.85, 95% CI, 0.73–0.99), family medicine physicians were associated with reduced use of mechanical ventilation (OR = 0.35, 95% CI, 0.19–0.62), and importantly, a trend of decreasing utilization after 2009 was noted for in-hospital resuscitation (OR = 0.76, 95% CI, 0.59–0.99). Finally, patients with ESRD and those provided with care by nephrologists were significantly more likely to receive dialysis treatments during EOL care.

## Discussion

This study extended the EOL literature on CKD care by leveraging the population-based research database to examine the costs and utilization of intensive services during EOL care. Elderly Taiwanese patients with CKD received a high intensity of EOL care, both in terms of medical spending and use of intensive health services, including surgery, ICU stay, mechanical ventilation, resuscitation, and dialysis. The intensity of EOL care is shaped by a myriad of patient, physician, and hospital characteristics, as well as changes in payment policy on hospice care.

On average, elderly patients with CKD experienced very high rates of surgical interventions (41%), ICU admission (40%), mechanical ventilation (45%), and resuscitation (15%) during the final month of life. In comparison, in the US Medicare population, 18% of the beneficiaries underwent surgical procedures [42], and 24% and 9% of patients with cancer were admitted to ICUs and received life-sustaining treatment [including intubation, feeding tube placement, and cardiopulmonary resuscitation (CPR)], respectively, during the last month of their life [43]. Moreover, studies in other countries on patients with cancer have revealed consistently lower EOL surgical rates [44, 45] and utilization of ICU, mechanical ventilation, and resuscitation [33, 46–48] than those observed in our study population. In Taiwan, patents with cancer also had lower EOL care intensity: 11% with ICU admission, 29% with mechanical ventilation and 11% with CPR [35]. Consequently, in Taiwan, compared with the average spending of > US$10,000 for patients with CKD during their final month, the corresponding costs were approximately US$1800 for lung cancer [49] and US$2000– US$2300 for patients with liver cancer [50]. Our findings are consistent with those of an earlier US study reporting that ESRD patients received a higher intensity of EOL care than did patients with cancer [24], even though the rates of intensive procedures observed in US ESRD populations were still lower than the estimates reported here in Taiwan [24, 31, 51].

**Fig. 1:**
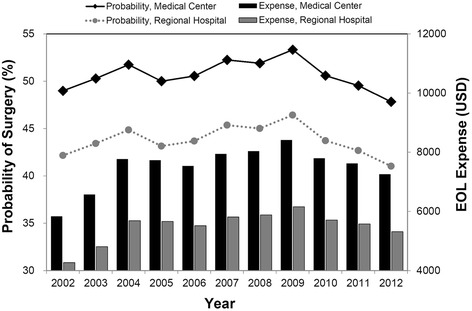
Predicted end-of-life expenses^a^ and probability of surgical interventions for average ESRD patients in Taiwan, 2002–2012.^b^ [^a^Converted at US$1 = NT$32.4; ^b^Estimates are based on linear predictions of multivariate log-linear and logistic models with year fixed effects with all variables, except ESRD and hospital accreditation, kept at the mean values.]; Abbreviations: ESRD, end-stage renal disease; EOL, end-of-life

Studies examining the long-term trend of EOL care intensity have typically identified a steady increase over time [6, 52], and the reduction in EOL care costs has always been a challenge for policy-makers [53]. Although recent studies in the US have indicated that hospice programs under Medicare are cost-saving [54, 55] and that geographical access to hospice care has greatly improved in the past decade [56], no signs of a decrease in the intensity and cost of EOL care were observed on a systematic scale [57, 58]. In our analysis, we also noted an overall trend of a 4% annual increase in costs. Nevertheless, with the 2009 NHI policy to reimburse providers for non-cancer hospice care, we detected a significant declining trend in costs and surgical rates between 2009 and 2012. For instance, for an average ESRD decedent in a medical center, inpatient expenses between 2009 and 2012 fell by 14%, and the probability of surgical interventions in 2012 was also lower than that in 2009 (48% and 53%, respectively; Fig. 1). The odds of receiving resucitation also showed a significant decline after 2009, despite the limited sample size used for the sub-sample analyses. In our sample, inpatient hospice care was not used until 2009, when it began to increase at a low rate to reach approximately 4% in 2012 (Appendix 2). Therefore, the impact of the policy might be due to not only the increased use of hospice care but also the increased awareness and change in practices of the providers. Because of the lack of a control group, we cannot claim definitive causality of the policy impact; however, all the consistent evidence suggests that payment intervention is likely to be an essential tool to promote desired changes in EOL care. Future studies to confirm this finding are warranted.

In addition to policy interventions, our findings also indicate that the characteristics of both the demand and supply sides play significant roles in the intensity of EOL care. Regarding patient characteristics, in addition to age, sex, and the presence of ESRD and comorbidities, which have all been repeatedly shown to play crucial roles [5–7, 42, 59–61], we also revealed a discrepancy across socioeconomic status (SES), which contradicted the negative findings of earlier US studies [62, 63]. Regarding general medical services, individuals of lower SES are typically treated similarly [64–66] or less intensively [67, 68]; however, we determined that they were likely to receive a higher intensity of EOL care. An earlier study on Taiwanese cancer decedents also reported that lower income was associated with more aggressive treatments [69]. Such inequality in EOL care across income groups is troubling because individuals and households with low SES are particularly vunerable to the potential financial harm caused by medical bills [34].

Our results also confirmed prior reports of the crucial roles of non-clinical provider characteristics in cancer EOL care [11, 33] and extended further to the CKD populations. For instance, our findings of higher EOL costs of care and odds of any surgical procedures at medical centers and regional hospitals are in agreement with the literature that facilities with higher bed capacities are often associated with higher EOL care intensities [6, 11, 12, 15, 70]. In addition, the effects of physician characteristics identified in this study also implied that education and training can potentially help in reducing costs and intensity of EOL care, which is consistent with the findings of previous studies examining internal medicine residency training and intensity of practice during EOL care [71]. First, elderly CKD decedents attended by older physicians had lower costs and odds of receiving surgical interventions or ICU admission during the last month of their life. Through training programs for younger professionals, an opportunity exists to encourage the practice of lower intensity care for EOL. Second, the intensity of care is strongly associated with the physician specialty and is particularly low in family medicine physicians. This provides the evidence supporting the inclusion of palliative care in medical training to lower EOL care costs.

Our findings should be generalized with caution, particularly considering the observational nature of the data. The association identified between various predictors and costs of EOL care could be influenced by other unobserved factors, especially when claims did not contain information on patient preferences. Meanwhile, our results among patients with CKD were consistent with those of prior studies, most of which investigated cancer care, providing some assurance of the validity. In addition, a constraint of our data is the inability to specify the primary reasons why the patients were admitted. However, by limiting our sample to the elderly, we tried to minimize influences from patient heterogeneity on remaining life expectancy. Third, given the data limitation, we were not able to distinguish patients with different CKD stages, except for those with ESRD. However, many patients with CKD are diagnosed at late stages (CKD 4 and 5) because of the asymptomatic nature of CKD [72] and systemic barriers to optimal care for late-stage CKD [73]. The heterogeneity in healthcare services across different CKD stages may be less obvious, and CCI scores may correlate better with healthcare needs than with CKD stage alone [74]. Although the variation and nature of EOL care intensity across different CKD stages warrants future studies, CKD stage should have very limited effects on biasing the estimates of other determinants assessed in this study. Finally, our examination of the utilization of ICU, mechanical ventilation, resuscitation, and dialysis was constrained by the limited sample size of the sub-sample. Although the coefficients across different models were consistent, many of them did not meet our threshold of statistical significance.

## Conclusions

EOL care intensity is an important and challenging issue, both in terms of the quality of care and cost containment. Our study of EOL care among elderly patients with CKD in Taiwan who died in hospitals or shortly after discharge demonstrated a high overall intensity, with significant variation in the manner of treatment during the last 30 days of life. The costs and odds of receiving intensive services were driven by a combination of physician characteristics, facility factors, and payment policies, as much as they were shaped by individual patient characteristics. These findings, together with the identified socioeconomic disparities, suggest that several opportunities exist to reduce EOL care costs, such as through payment intervention and education, to create responsive health systems that provide a high value of care to this vulnerable population.

## Figures and Tables

**Fig. 2:**
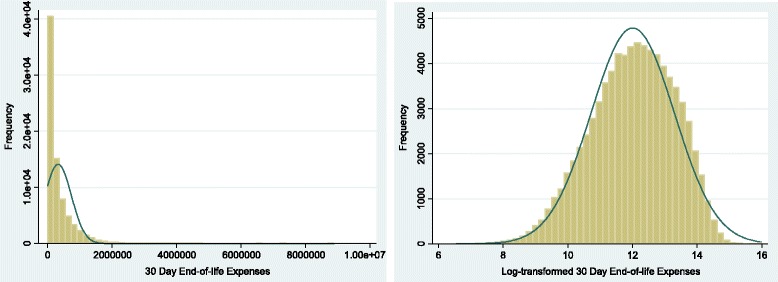
Distribution of 30-day end-of-life expenses, before and after log transformation

## Appendix 2

**Table 5:** Inpatient hospice care use in the study cohort

Year	Number of User	% of Total Patient Population	Total Number of Patients
2002	0	0.0%	4989
2003	0	0.0%	7164
2004	0	0.0%	6917
2005	0	0.0%	6236
2006	0	0.0%	6156
2007	0	0.0%	5934
2008	0	0.0%	5747
2009	31	0.6%	5295
2010	165	3.0%	5544
2011	228	4.3%	5289
2012	211	3.6%	5853
Total	635	1.0%	65,124

## Abbreviations

CCI: Charlson Comorbidity Index
CI: Confidence interval
CKD: Chronic kidney disease
CPR: Cardiopulmonary resuscitation
EOL: End-of-life
ESRD: End-stage renal disease
ICU: Intensive care unit
NHI: National Health Insurance
NHIRD: National Health Insurance Research Database
OR: Odds ratio
SD: Standard deviation
SES: Socioeconomic status
